# Combined effect of laser acupuncture and electroacupuncture in knee osteoarthritis patients: A protocol for a randomized controlled trial

**DOI:** 10.1097/MD.0000000000019541

**Published:** 2020-03-20

**Authors:** Szu-Ying Wu, Chien-Hung Lin, Nai-Jen Chang, Wen-Long Hu, Yu-Chiang Hung, Yu Tsao, Chun-En Aurea Kuo

**Affiliations:** a Department of Chinese Medicine, Kaohsiung Chang Gung Memorial Hospital and Chang Gung University College of Medicine, Kaohsiung; b Department of Nursing, Meiho University, Pingtung; c Department of Sports Medicine; d College of Medicine, Kaohsiung Medical University; e Fooyin University College of Nursing; f School of Chinese Medicine for Post Baccalaureate, I-Shou University; g College of Management, National Kaohsiung University of Science and Technology; h Department of Leisure and Sports Management, Cheng Shiu University, Kaohsiung, Taiwan.

**Keywords:** electroacupuncture, knee osteoarthritis, laser acupuncture, low-level laser therapy, photobiomodulation

## Abstract

**Background:**

Knee osteoarthritis (KOA) is a common degenerative joint disorder that affects 250 million people globally. KOA can lead to disability and is often associated with cardiovascular disease, poor quality of life, and mortality. The most common treatment for KOA is non-steroidal anti-inflammatory drug administration. However, the analgesic effect is limited and often accompanied by multiple side effects. Hence, many KOA patients opt for complementary and alternative medicine. Acupuncture is one of the most popular complementary treatments with great analgesic effect and minimal side effect. Electroacupuncture (EA) and laser acupuncture (LA) have been known to reduce pain in KOA patients. However, to date, no study has assessed the benefits of combining these two therapies.

**Methods:**

Fifty participants diagnosed with KOA, aged 50 years or older, and with consistent knee pain for more than 3 months were recruited and randomly assigned to the treatment group (EA plus LA) or control group (EA plus sham LA without laser output). All subjects in the treatment group will undergo a combined EA and LA treatment thrice a week for 4 weeks. The acupuncture will be performed on GB33, GB34, SP9, SP10, and ST36 sites. The treatment group will receive acupuncture with a transcutaneous electrical nerve stimulator at GB33, GB34, SP9, and SP10 sites and with LA at EX-LE5, ST35, and BL40 sites. The subjects in the control group will undergo the same treatment modality as the treatment group, except these subjects will not be exposed to laser output. Outcome measurements will include visual analog scale, Western Ontario McMaster Universities Osteoarthritis Index, Knee injury and osteoarthritis outcome, body composition analysis, knee range of motion, quadriceps muscle stiffness, one-leg standing with eyes open test, and the 30-s chair stand test before and after 4 weeks of intervention.

**Objectives:**

This protocol aims to investigate the combined effect of EA and LA in KOA patients.

## 1 Introduction

Knee osteoarthritis (KOA) is a common degenerative joint disorder that primarily affects elderly adults.^[1]^ Approximately 250 million people are affected by KOA globally, and this number is expected to increase in the future.^[2]^ KOA symptoms include knee stiffness, arthralgia, and limitation in range of motion, which can lead to a decrease in quality of life and even disability in severe cases.^[3,4]^ A previous study showed that KOA also affects the structure of the knee joint.^[5]^ Synovial inflammation, abnormal bone structure, and bone marrow lesions are commonly observed in KOA patients, which could be the underlying cause of persistent knee pain.^[5]^ Among contributors to global disability, KOA ranks 11th, and some of its secondary diseases include obesity, cardiovascular disease, and leading to higher mortality.^[6]^

The modality of KOA treatment depends on the severity of its clinical symptoms. Currently, non-pharmacological interventions such as exercise and weight loss are the main treatment modalities for mild KOA.^[7]^ In cases when such non-pharmacological interventions are inadequate, patients are often treated with topical non-steroidal anti-inflammatory drugs (NSAIDs).^[8]^ In moderate to severe cases, the treatment modalities include exercise,^[9]^ knee braces,^[10]^ walking aids,^[11]^ oral NSAIDs,^[12]^ and surgery.^[13]^ Oral NSAIDs are commonly used medicine to alleviate KOA-associated pain; however, long-term use of NSAIDs is often associated with cardiovascular diseases such as myocardial infarction, kidney injury, and gastrointestinal symptoms such as gastrointestinal bleeding.^[14,15]^ Therefore, alternative treatment modalities to alleviate KOA symptoms are in high demand. Non-pharmacological KOA intervention includes exercise, heat and cooling packing, neuromuscular electrical stimulation, transcutaneous electrical nerve stimulation, low-level laser therapy (LLLT), massage, and acupuncture.^[16]^ The exercise programs emphasize on improving aerobic capacity, quadriceps muscle strength, and lower extremity performance.^[17]^

Acupuncture is one of the most popular treatment modalities to alleviate KOA symptoms. Transcutaneous electrical nerve stimulation and acupuncture have similar mechanisms to relieve pain without serious adverse effects.^[18]^ Electroacupuncture (EA), a combination of acupuncture and transcutaneous electrical nerve stimulation, has become a popular treatment for KOA and is associated with a low risk of adverse reaction.^[19–21]^

Laser acupuncture (LA), a form of LLLT, is painless, non-invasive, has no risk of infection, and can be combined with traditional acupuncture.^[22,23]^ Currently, LA is primarily used to address arthralgia or muscular pain.^[24,25]^ LLLT has been reported to be a cost-effective analgesic therapy.^[26]^ However, the benefits of LA in alleviating KOA symptoms have remained elusive. In one previous clinical trial, LLLT has been suggested to alleviate KOA symptoms by increasing and decreasing serum beta-endorphin and substance P levels, respectively.^[27]^ Some studies analyzing the effect of LLLT in KOA patients produced ambiguous results. A meta-analysis on these studies conducted by Stausholm et al^[28]^ showed that LLLT can reduce pain and address disability in KOA patients. A randomized controlled trial conducted by Alghadir et al showed that LLLT was effective in reducing knee pain intensity at rest and movement, function, and ambulation. Therefore, LLLT seemed to be an effective modality for short-term pain relief and function improvement in patients with chronic knee OA.^[29]^

To date, there are no studies on the effect of a combined application of EA and LA in KOA patients. We will conduct a two-arm parallel clinical trial. This study aims to investigate the effect of a combined application of EA and LA and explore the effects of LA in terms of knee pain, knee range of motion, knee stiffness, and quadriceps strength and function in KOA patients. We hypothesize that a combination of LA and EA will produce more beneficial results than the application of these therapies alone in addressing KOA symptoms.

## 2 Methods

### 2.1 Ethics approval

The study was approved by the Human Ethics Committee of Chang Gung Medical Foundation Institutional Review Board, IRB No. 201901018A3C501. All participants will provide written informed consent before their enrollment in this study. Personal information will be collected, shared, and maintained in an independent closet to protect confidentiality before, during, and after the trial. The protocol has been registered at ClinicalTrials.gov (Identifier: NCT04188925).

### 2.2 Study design

**Figure 1:**
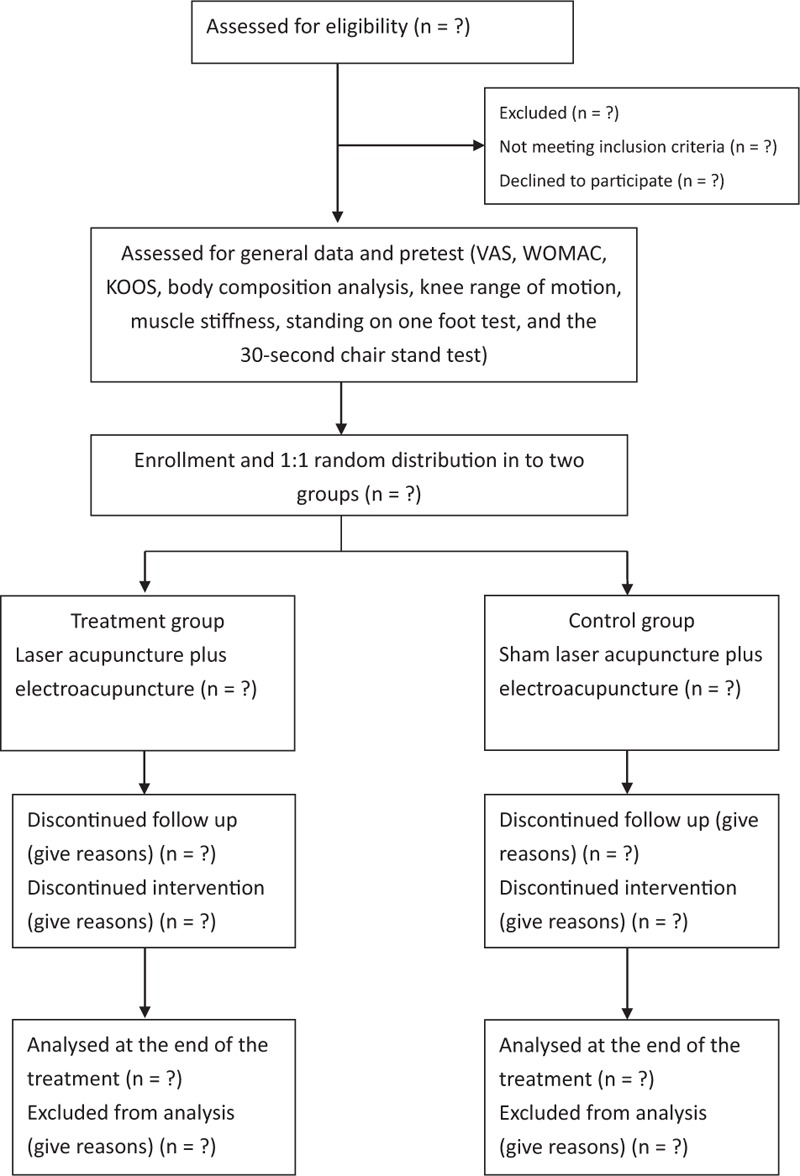
Flowchart of the trial. KOOS = Knee injury, and osteoarthritis outcome, VAS = Visual analog scale, WOMAC = Western Ontario McMaster Universities Osteoarthritis Index.

This randomized controlled trial will be conducted at the Department of Chinese Medicine at the Kaohsiung Chang Gung Memorial Hospital from August 2019 to December 2020. The participants recruited from our outpatient departments will be randomly allocated to the treatment group (LA plus EA, n = 25, expected) or the control group (sham LA without laser output plus EA, n = 25, expected). All participants will receive 12 sessions of treatment over 4 weeks. The study design is depicted in Figure 1.

### 2.3 Participants

The diagnosis of KOA depends on the patient's history (age more than 50 years old, persistent knee pain, limited morning stiffness, reduce function, crepitus, restricted movement and bony enlargement)^[30]^ and radiography (osteophytes formation, joint space narrowing, subchondral bone sclerosis, and subchondral cysts).^[31]^ In this study, we recruited patients who were diagnosed with KOA, aged more than 50 years old and had knee pain for more than 3 months. Designated physicians evaluated the eligibility of all participants. Patients’ inclusion criteria for this study were based on the following factors: visual analog scale (VAS) > 3 points in daily activity, Kellgren–Lawrence Grading Scale grade is between 1 and 3 on knee X-ray, and informed consent obtained adequately. Patients’ exclusion criteria for this study were based on the following factors: history of knee arthroplasty, traumatic injury related joint deformity, body mass index more than 35, pacemaker insertion, photosensitivity, intra-articular injection or acupuncture 1 month before the enrolment, and any factors that may affect the evaluation of the outcome, such as psychiatric disorder and moderate to severe mental retardation.

### 2.4 Sample size and randomization

The necessary sample size was calculated from the result of a previous study on the effect of LLLT. In this study conducted by Al Rashoud et al, the mean difference (standard deviation) of the VAS at the baseline before the treatment were 6.4 (1.9) at the treatment group, and 5.9 (1.8) at the control group, and the mean difference (standard deviation) of the VAS after completing the treatment were 3.2 (1.7) at the treatment group, and 3.8 (2.3) at the control group.^[32]^ We aimed to achieve an effect size of 0.569, a significance level (α) of 0.05, the desired power (1 − β) of 0.80, and correlation among repetition measures of 0.5, and as such, the estimated sample size for this study was 22. Anticipating a 20% dropout rate, a total of 50 participants were recruited.

We used a randomized block design to distribute participants equally into two groups, A and B, with the help of a research randomizer (https://www.randomizer.org). Designated physicians enrolled participants who met the inclusion criteria and grouped the participants according to the result of randomizer by the website. The trial participants, outcome assessors, and data analyst will be blinded after assignment to interventions using labels A and B for the 2 groups.

### 2.5 Intervention

After signing the inform consent, participants in both groups will receive a pretest to obtain information on the frequency of analgesic used, the VAS during rest and activity, Western Ontario McMaster Universities Osteoarthritis Index (WOMAC), Knee injury and Osteoarthritis Outcome Score (KOOS), body composition analysis, knee range of motion test, quadriceps muscle stiffness, one-leg standing with eyes open test, and the 30-s chair stand test.

**Figure 2:**
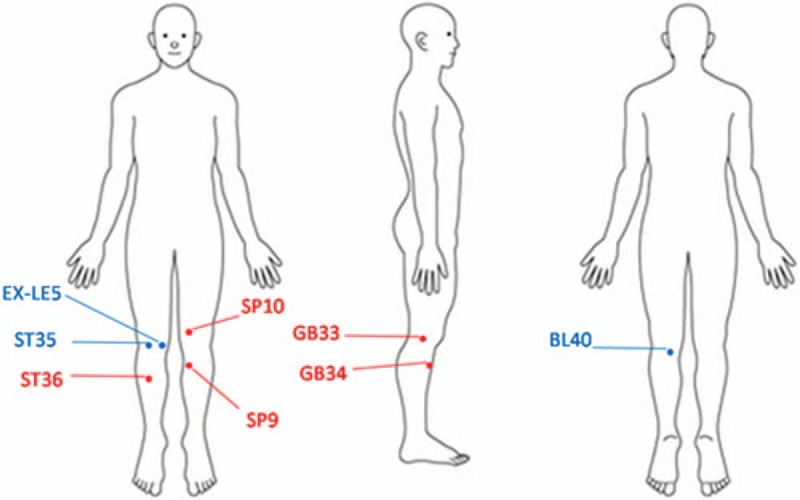
Acupoints used for knee osteoarthritis in this study. The blue color acupoints correspond to interventions with laser acupuncture, whereas the red color acupoints correspond to intervention with acupuncture.

**Figure 3:**
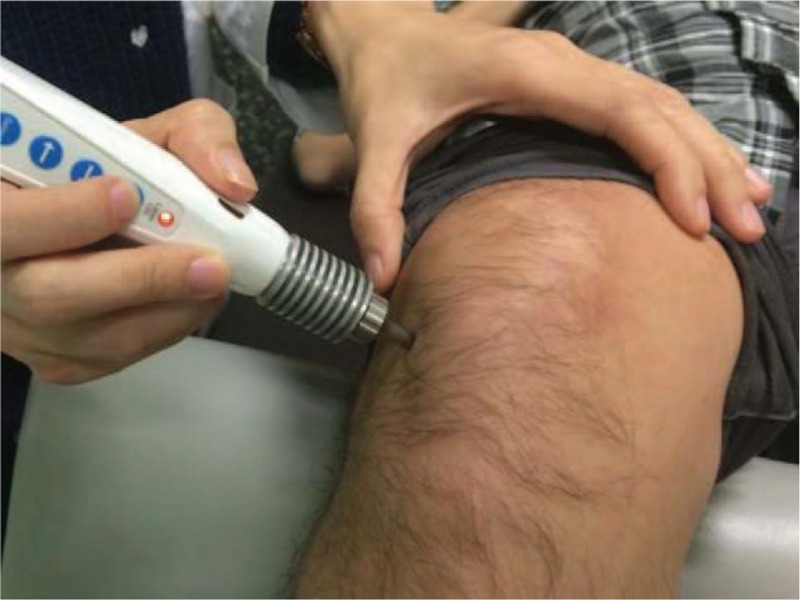
Laser acupuncture performed at EX-LE5 acupoint by the LaserPen device.

The study participants will receive 12 sessions of LA and EA (the treatment group) or sham LA and EA (the control group) over 4 weeks. LA will be performed using a gallium aluminum arsenide LaserPen (maximal power, 150 mW; wavelength, 810 nm; area of probe, 0.03 cm^2^; power density, 5 W/cm^2^; and pulsed-wave; RJ-Laser, Reimers & Janssen GmbH, Waldkirch, Germany). The LA treatment will be applied to EX-LE5, ST35, and BL40 (Fig. 2) for 80 s, at 150 mW, to deliver a total treatment dose of 6 J/cm^2^ (Fig. 3). An experienced physician who has adequate training and a license in practicing Chinese medicine in Taiwan for more than 7 years will perform all LA treatments. Both the physician and patients will wear necessary protective gear. The control group will undergo the same procedure as the treatment group but with an inactive LaserPen (with the indicative light on but no laser output). Both of the LaserPen in two groups are labeled with A and B, and the physical appearance of both LaserPens will be the same to eliminate any bias from physicians and the participants.

**Figure 4:**
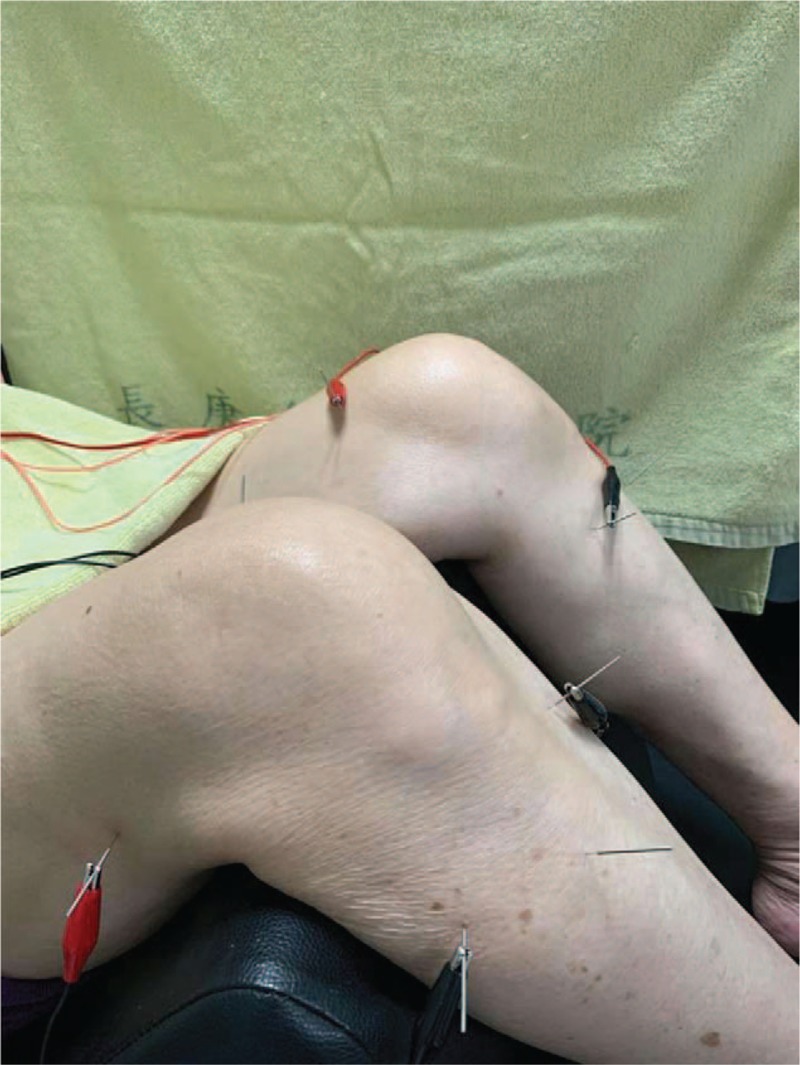
Electroacupuncture performed at GB33, GB34, SP9, and SP10 acupoints by transcutaneous electrical nerve stimulator.

EA is a combination of acupuncture and transcutaneous electrical nerve stimulator. The transcutaneous electrical nerve stimulator device is produced by the Ching Ming Medical Device Company. This machine (ID number 001147) is verified by the Ministry of Health and Welfare in Taiwan. The acupuncture will be first performed on GB33, GB34, SP9, SP10, and ST36 sites (Fig. 2). Subsequently, transcutaneous electrical nerve stimulator will be used on the GB33, GB34, SP9, and SP10 sites. There are electrical outputs in a transcutaneous electrical nerve stimulator, and each electric output has two lines (black and red) representing the positive and negative pole, respectively (Fig. 4). When both electrical outputs are connected to paired needles, it produces electric currents to stimulate the specific acupoints. The electric wave mode will be continuous wave to achieve stable stimulation, at a frequency of 2 Hz for 15 min. The power of the electric current will be adjusted to make each patient feel the electric stimulation but still comfortable during the treatment process. All the acupoints are selected and localized according to the guideline of WHO Standardized Acupuncture Point Location.^[33]^

After treatments in each session, a designated physician will record the frequency of analgesic used, the VAS during rest and activity, the response to our therapy, and any adverse events. The patients can receive analgesics during the treatment period; however, they will be prohibited from receiving other acupuncture, rehabilitation program or knee surgery in any other clinic. If the patients do not follow these provisions, we will exclude these patients from our study protocol. On successful completion of 12 sessions, all participants will be subjected to the same exam and questionnaire as during the pretest.

### 2.6 Outcome measurements

We will evaluate the KOA patients with subjective tests and objective questionnaires at the beginning of the treatment and after 12 weeks of therapy. The data will be collected and entered into the patient's medical record in a computer, and all patient-related documents will be securely stored. Only the study-related investigators, including the physicians and the assistants will be allowed to access to the trial dataset. The primary outcomes will be the difference in the frequency of analgesic used and VAS scores during rest and activity between the treatment group and the control group. The secondary outcomes will be the difference between the control and treatment groups in terms of WOMAC, KOOS, body composition analysis, knee range of motion test, quadriceps muscle stiffness, one-leg standing with eyes open test, and the 30-s chair stand test after completing the 12 sessions of treatments.

We will evaluate the frequency of analgesic used to treat knee pain after each intervention, including acetaminophen, a non-steroidal anti-inflammatory drug, or opioid drug. VAS will be used to evaluate the intensity of knee pain during activity and rest on a scale of 0 to 10, corresponding to the minimum and maximum discomfort intensity. The minimal clinical important difference (MCID) of VAS is 1.8 units,^[34]^ implying that an effective treatment will improve the VAS score by 1.8 points. WOMAC is a subscale for assessing the physical function of knee joints. There will be 17 questions with grading from 0 to 4 points for each question. Thus, the total score will be between the ranges of 0 to 68 points. Higher points correspond to more difficulties in performing a given activity. The MCID of WOMAC is 6 units.^[35]^ The KOOS is a self-administered scale and evaluates five outcomes, including pain, symptoms, activities of daily living, sport and recreation function, and knee-related quality of life. The scale is divided into five factions, including none, mild, moderate, severe, and extreme, which represent the severity of the deterioration of knee function and the impairment in daily living.^[36]^ A previous study showed that one-time LLLT had some effects on improving the knee function and muscle strength of quadriceps in KOA.^[37]^ We use Myoton-3 myometer and InBody 270 to measure muscle functions. In Myoton-3 myometer, the tip is placed on the midpoint of the patient's rectus femoris to measure muscle stiffness, representing the muscle ability to resist the changes of its shape, caused by external forces.^[38]^ InBody 270 can measure body composition, including the fraction of muscle and fat, and the muscle mass distribution of four limbs. This device will be used to evaluate the composition of thigh muscles and any possible muscle imbalance. One-leg standing with eyes open test will be used to assess balance and muscle strength of lower extremities.^[39]^ The patient will stand on one leg with their hands on hips and eyes opening for up to 120 s. We will record the time that the patient can stand on one leg under the instruction. Another functional assessment is the 30-s chair stand test, which can evaluate the muscle performance and the endurance of lower limbs.^[40]^ The patient will sit on the chair with bilateral feet stepping on the ground and hands crossed over the chest. Subsequently, the patient stands up and then sits down. Completing one standing up and then sitting down scores one, etc. We will calculate the number of times the patient can stand up and then sit down in 30 s. A designated study assistant will evaluate these tests.

The reason for patients who are not completing the follow-up visit or dropping out of this study, such as adverse events, suboptimal response to therapy, failure to return for following up, and refusal to receive treatment will be recorded. The potential adverse events will also be recorded although only 6 J/acupoint will be delivered to each acupoint.

### 2.7 Statistical analysis

All data will be presented in the form of mean ± standard deviation. The independent *t* test and chi-square tests will be used to assess and compare the baseline patient characteristics between the treatment and the control groups. Repeated measure analysis of variance and the generalized estimating equation will be used to evaluate changes in the VAS, WOMAC, KOOS, body composition, knee range of motion, rectus femoris stiffness, standing on one foot, and 30-s chair stand test between the treatment and the control groups. Statistical significance will be considered at a *P*-value of <.05. All statistical analyses will be performed with the SPSS for Windows, version 26 (Statistics 26, SPSS, IBM Corp., Chicago, IL).

### 2.8 Data monitoring

The IRB and Kaohsiung Chang Gund Memorial Hospital's statisticians will be a part of the data monitoring committee in this study. LA and EA are general practices. LA is a routine intervention and non-invasive treatment. The common side effects of EA are subcutaneous bleeding, pain, fainting during acupuncture treatment, while in very rare cases, serious complications such as pneumothorax or septicemia are possible.^[41]^ However, since the designated acupuncture physicians are well trained in aseptic acupuncture procedures and proficient in human anatomy, the risk of serious complications is minimal. Additionally, the risk of fainting during the EA treatment will be extremely low because, in the study setting, the patient will be treated in a supine position.

## 3 Discussion

The prevalence of KOA in 50 years or older population is about 37% in Taiwan. This frequency is expected to increase, as Taiwan has a large number of elderly adults.^[2,42]^ According to Taiwan's National Health Insurance Research Database, the frequency of KOA patients requiring total knee replacement has increased between 1996 and 2010. Also, the cost associated with total knee replacement has increased from $39.69 to $83.35 million.^[42]^ As such, it is important to identify cost-effective therapy for KOA; EA and LA are known cost-effective analgesic treatments.^[26,43]^

Berman et al conducted a large randomized controlled trial study in 2004, which recruited 570 KOA patients and showed that EA had positive effects on improving knee function and relieving pain in KOA patients.^[19]^ EA cannot only alleviate pain and improve stiffness and disability, but also lead to a significant rise and fall in plasma beta-endorphin and plasma cortisol, respectively, after 10 sessions of treatment. Collectively, these effects could be the underlying analgesic mechanism of EA.^[44]^ This hypothesis is further validated by the fact that EA with etoricoxib was more effective than either placebo plus etoricoxib or etoricoxib alone in addressing KOA symptoms.^[43]^ Recently, a systematic review suggested that the therapeutic sessions of EA for at least 4 weeks significantly improved the VAS, WOMAC, and Lysholm knee score scale (LKSS).^[45,46]^ A detailed literature review on the application of EA in KOA patients, including treatment frequency and assessment, is listed in Table 1.

**Table 1:**
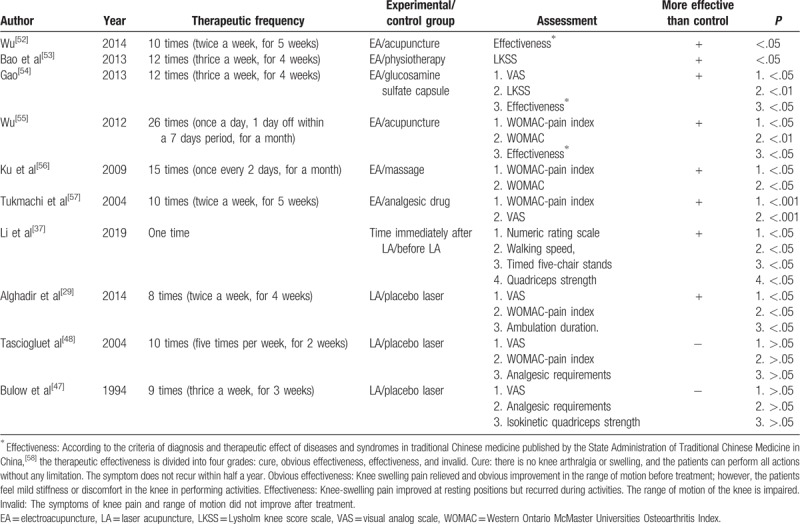
Literature review on the effectiveness of electroacupuncture or laser acupuncture in knee osteoarthritis.

A double-blind, randomized control trial in 1994 used a low power Ga–Al–As laser treatment in KOA patients at a frequency of 3 times per week for 3 weeks and showed that there was no difference in results between the treatment group and the control group.^[47]^ The study concluded that a placebo effect was observed since the symptoms improved before and after interventions in both the treatment group and the control group.^[47]^ Another double-blind, randomized trial in 2004 with LLLT (50 mini-watt, 830 nm, and output energy of 1.5 or 3 J/point) at a frequency of 10 times in 2 weeks showed that there was no significant improvement in VAS and WOMAC parameters between the treatment group and control groups.^[48]^ A meta-analysis showed that LA had some positive effects on the WOMAC parameter in KOA patients; however, insufficient information on wavelength, energy density, and treatment duration made results’ interpretation difficult.^[49]^ In a recent meta-analysis, LLLT at a strength of 4 to 8 J and 785 to 860 nm wavelength with the mean duration of the treatment periods 3.53 weeks was found to reduce pain and disability in KOA patients after the end of therapy and during follow-ups 3 months.^[28]^ The multi-focal LLLT on quadriceps had shown not only analgesic effect but also improved function and muscular strength in KOA patients.^[37]^ The reasons that these studies exhibited ambiguous results may be the differences in laser dosage, laser power density, the treatment sites, and the treatment frequencies.^[50]^

Among many of the mechanisms of KOA, synovial inflammation (synovitis), abnormal bone structure, osteophytes, and bone marrow lesions can result in knee pain.^[5]^ It is possible that EA treatments could lead to activation of the nervous system and inhibition of inflammatory and neuropathic pain by desensitizing peripheral nociceptors and reducing pro-inflammatory cytokines.^[51]^ The clinical effects of LA are likely to encompass anti-inflammatory, neural modulation, cell healing and regeneration effects.^[23]^ Both LA and EA have anti-inflammatory and neural modulation, which may decrease knee pain in KOA. Conversely, exercise therapy can reduce knee pain in patients with KOA and these exercise therapies are focused on improving aerobic capacity and quadriceps muscle strength.^[17]^ LA has an analgesic effect and can improve the function and muscle strength of quadriceps in KOA.^[37]^ Besides, LA and EA have many advantages, including low risks of complications, pain, cost, and high analgesic efficacy. Although some studies confirmed such beneficial effects of LA or EA in improving KOA symptoms, the combined effect of EA and LA on KOA remains elusive.

In this study protocol, we will use LA at 6 J/acupoint delivered with an 810 nm laser in addition to EA treatment for 4 weeks. LA treatments are poised to heal damaged chondrocytes, which are more susceptible to pain stimulation.^[59]^ The outcome evaluations will include analyses of subjective symptoms, quadriceps muscle functions, and body composition measurements. In conclusion, we hypothesize that a combination of LA and EA is expected to be more effective than these therapies alone in alleviating knee pain and stiffness and increasing muscle strength of lower limbs, which can further improve the balance and quality of life. Moreover, this combined therapy may also benefit in reducing the need for total knee replacement therapy and further decrease the cost coverage of national health insurance.

## Acknowledgments

The authors would like to extend their appreciation to all the patients who participated in this study. We appreciate the assistance with the statistical analysis provided by the Biostatistics Center, Kaohsiung Chang Gung Memorial Hospital.

## Author contributions

SYW and NJC conceived, designed, and planed the study. SYW, CEK and WLH are recruiting the study participants and performing the interventions. WLH and YCH supervised the study. SYW, CHL, YT and CEK will interpret and analyze the data. CHL and SYW drafted the manuscript. CEK critically revised the manuscript for important intellectual content. All authors have full access to the manuscript and take responsibility for the study design. All authors have approved the manuscript and agree with submission.

Chun-En Aurea Kuo: 0000-0002-1563-8651.

Chun-En Aurea Kuo orcid: 0000-0002-1563-8651.
